# Upright, prone, and supine spinal morphology and alignment in adolescent idiopathic scoliosis

**DOI:** 10.1186/s13013-017-0111-5

**Published:** 2017-02-22

**Authors:** Rob C. Brink, Dino Colo, Tom P. C. Schlösser, Koen L. Vincken, Marijn van Stralen, Steve C. N. Hui, Lin Shi, Winnie C. W. Chu, Jack C. Y. Cheng, René M. Castelein

**Affiliations:** 1 Department of Orthopaedic Surgery, University Medical Center Utrecht, P.O. Box 85500, 3508 GA Utrecht, The Netherlands; 2 Image Sciences Institute, University Medical Center Utrecht, Utrecht, The Netherlands; 3 Imaging Division, University Medical Center Utrecht, Utrecht, The Netherlands; 4 Department of Imaging and Interventional Radiology, Prince of Wales Hospital, The Chinese University of Hong Kong, Shatin, Hong Kong; 5 Department of Diagnostic Radiology and Organ Imaging, Prince of Wales Hospital, The Chinese University of Hong Kong, Shatin, Hong Kong; 6 Department of Orthopaedics and Traumatology, Prince of Wales Hospital, The Chinese University of Hong Kong, Shatin, Hong Kong

**Keywords:** Adolescent idiopathic scoliosis, Three-dimensional morphology, Body positioning, Upright radiographs, Computed tomography, Magnetic resonance imaging

## Abstract

**Background:**

Patients with adolescent idiopathic scoliosis (AIS) are usually investigated by serial imaging studies during the course of treatment, some imaging involves ionizing radiation, and the radiation doses are cumulative. Few studies have addressed the correlation of spinal deformity captured by these different imaging modalities, for which patient positioning are different. To the best of our knowledge, this is the first study to compare the coronal, axial, and sagittal morphology of the scoliotic spine in three different body positions (upright, prone, and supine) and between three different imaging modalities (X-ray, CT, and MRI).

**Methods:**

Sixty-two AIS patients scheduled for scoliosis surgery, and having undergone standard pre-operative work-up, were included. This work-up included upright full-spine radiographs, supine bending radiographs, supine MRI, and prone CT as is the routine in one of our institutions. In all three positions, Cobb angles, thoracic kyphosis (TK), lumbar lordosis (LL), and vertebral rotation were determined. The relationship among three positions (upright X-ray, prone CT, and supine MRI) was investigated according to the Bland-Altman test, whereas the correlation was described by the intraclass correlation coefficient (ICC).

**Results:**

Thoracic and lumbar Cobb angles correlated significantly between conventional radiographs (68° ± 15° and 44° ± 17°), prone CT (54° ± 15° and 33° ± 15°), and supine MRI (57° ± 14° and 35° ± 16°; ICC ≥0.96; *P* < 0.001). The thoracic and lumbar apical vertebral rotation showed a good correlation among three positions (upright, 22° ± 12° and 11° ± 13°; prone, 20° ± 9° and 8° ± 11°; supine, 16° ± 11° and 6° ± 14°; ICC ≥0.82; *P* < 0.001). The TK and LL correlated well among three different positions (TK 26° ± 11°, 22° ± 12°, and 17° ± 10°; *P* ≤ 0.004; LL 49° ± 12°, 45° ± 11°, and 44° ± 12°; *P <* 0.006; ICC 0.87 and 0.85).

**Conclusions:**

Although there is a generalized underestimation of morphological parameters of the scoliotic deformity in the supine and prone positions as compared to the upright position, a significant correlation of these parameters is still evident among different body positions by different imaging modalities. Findings of this study suggest that severity of scoliotic deformity in AIS patients can be largely represented by different imaging modalities despite the difference in body positioning.

## Background

Adolescent idiopathic scoliosis (AIS) is a complex three-dimensional (3-D) deformity of the spine, with a prevalence of 1.5–3% within the general population, that normally develops in the beginning of the growth spurt of previously healthy adolescents [1, 2]. For diagnosis, monitoring of progression, and clinical decision-making, periodical radiographic follow-up is traditionally performed using posterior-anterior and lateral upright radiographs. The Scoliosis Research Society defines scoliosis as a lateral curvature of the spine of more than 10° in the coronal plane on upright radiographs, also emphasizing the importance of radiography [3]. In addition, supine or prone magnetic resonance imaging (MRI) and computed tomography (CT) are frequently used to obtain more in-depth information about neuroaxis and bony architecture abnormalities. Some imaging involves ionizing radiation, and the radiation doses are cumulative, resulting in 9 to 10 times more radiation exposure and a 17 times higher incidence of cancer in the AIS cohort as compared to the general population [4, 5]. The importance of the 3-D character of the scoliotic deformity has long been recognized, and the upright X-ray, the gold standard, is not able to accurately represent the true 3-D deformity [6–9]. CT scanning can obtain accurate 3-D information of bony structures but relies on radiation and is not obtained upright [10]. An important step in attempts to visualize this 3-D character has been the development of low-dose upright imaging modalities that allow for 3-D reconstruction such as the EOS apparatus. Alternatively, MRI utilizes no harmful radiation but is considered inferior in visualizing the bone and is usually also not obtained upright. This study was designed to compare the morphology of the scoliotic spine on conventional radiographs in the upright position to those on MRI and CT obtained in supine and prone positions, respectively.

## Methods

### Study population

A subsequent series of AIS patients of ten or more years of age scheduled for scoliosis surgery in one of our centers between 2011 and 2014 and had complete standard pre-operative work-up were included in this study. Complete work-up consisted of posterior-anterior and lateral upright radiographs of the spine, supine bending X-rays, T2-weighted MRI (3.0-T MR scanner (Achieva TX; Philips Healthcare, Best, The Netherlands)) of the spinal cord for exclusion of neural axis abnormalities obtained in a supine position, and high-resolution CT (64 Slice Multi-detector CT scanner, GE Healthcare, Chalfont, St. Giles, UK, slice thickness 0.625 mm), obtained in a prone position. The CT scans were made for navigation purposes according to protocol in one of our institutions, in a position mimicking the position at surgery as closely as possible. Children with other spinal pathology than AIS, early onset scoliosis, previous spinal surgery, neurological symptoms or neural axis abnormalities, syndromes associated with disorders of growth, or atypical left convex thoracic curves or right convex (thoraco)lumbar curves were excluded to obtain an as homogeneous a population as possible. Moreover, cases that had undergone the different imaging methods with an interval of more than 6 months in between imaging were also excluded. Curve characteristics (curve type according to the Lenke classification, Cobb end vertebrae, and apical levels) were determined on the conventional radiographs [11, 12].

### Outcome parameters

The conventional radiographs were analyzed for main thoracic and (thoraco)lumbar Cobb angle, apical rotation (using Perdriolle’s method [13]), thoracic kyphosis (TK; superior endplate T4–inferior endplate T12), and lumbar lordosis (LL; superior endplate L1–sacral plate), using our picture archiving and communications system (PACS) workstation (Carestream solution working station, Carestream Health, Version 11.0, Rochester, NY, USA).

**Fig. 1:**
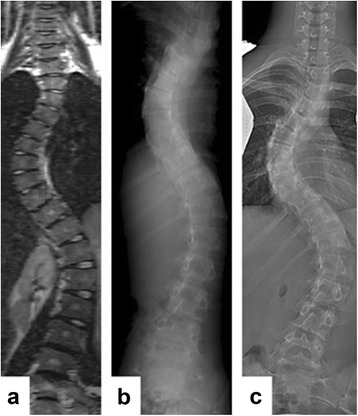
On the MRI and CT images, the main thoracic and (thoraco)lumbar Cobb angle, thoracic kyphosis, and lumbar lordosis were measured using the same technique as for the conventional radiographs on the image where the curve and endplates were best visible by using the multiplanar reconstruction (MPR, **a**) for the MRI and the digitally reconstructed radiograph (**b**) for the CT scan. **c** The conventional X-ray

**Fig. 2:**
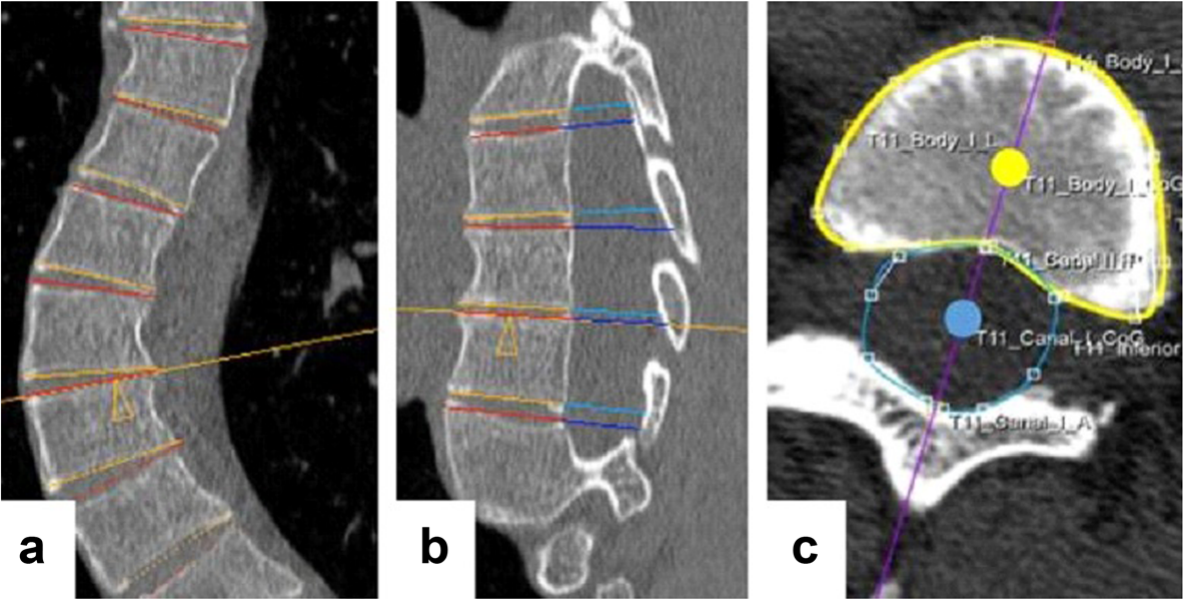
The orientation of the upper and lower endplates of each individual vertebra of the computed tomography scans was determined by using the semi-automatic software, correcting for coronal and sagittal (**a** and **b**) tilt, to reconstruct the true transverse sections. The observer drew a contour around the vertebral body (*yellow line* in **c**) and spinal canal (*blue line* in **c**). The software calculated a center of gravity of the vertebral body (*yellow dot* in **c**) and spinal canal (*blue dot* in **c**). For each endplate, its longitudinal axis was calculated as the line between those two points (*purple line* in **c**). The rotation of this axis minus the rotation of the neutral sacral plate represents the rotation of the endplate

On the MRI and CT images, the main thoracic and (thoraco)lumbar Cobb angle, TK, and LL were measured using the same technique as for the conventional radiographs, by using multiplanar reconstruction technique through the midsection of each vertebral body for the MRI and the digital reconstructed radiograph (DRR) for the CT scan (Fig. 1). The same levels were used for each patient on the three different imaging methods. Cobb end vertebrae were selected on the radiographs and applied to the other imaging modalities [14]. For measurement of apical rotation on the MRI and CT scans, complete 3-D reconstructions were acquired using semi-automatic analysis software (ScoliosisAnalysis 4.1, Imaging Division, Utrecht, The Netherlands) and a previously validated imaging method [15]. The observer selected the upper and lower endplates of the vertebral body. Then, the observer used the sagittal and coronal orientation of the endplates to correct for coronal and sagittal tilt. Thus, each vertebral level was manually positioned in the true transverse plane as accurately as possible. Subsequently, for each endplate, its longitudinal axis was calculated automatically after manual segmentation of the vertebral body and spinal canal. The rotation was defined as the rotation of this axis minus the rotation of the neutral sacral plate (Fig. 2).

Intra- and interobserver reliability for measurement of apical rotation using this method was tested in a previous study; intraclass correlation coefficients were 0.92 (95% confidence interval, 0.82–0.97) and 0.89 (0.74–0.95) on the 3-D scans [9]. In this study, the intra- and interobserver reliability analysis of the rest of the outcome parameters (Cobb angles, TK, and LL on all the three modalities and the vertebral rotation on the X-rays) was studied. Two observers independently analyzed a randomly selected subset of ten X-rays, CT scans, and MRI scans of the subjects.

### Statistical analysis

Statistical analyses were performed using SPSS 22.0 for Windows (SPSS Inc., Chicago, IL, USA). Descriptive statistics were computed providing means, ranges, and standard deviations. Potential outliers were identified. The agreement between the three positions was tested according to the Bland-Altman plot; first, the one-sample *t* test showed if there was a significant difference between the measurements; second, if there was no significant difference, the regression analysis showed if there was agreement between the measurements [16]. The two-way mixed intraclass correlation coefficient (ICC) was used to evaluate the correlation between the parameters in different body positions. The intra- and interobserver reliability were obtained as intraclass correlation coefficients. The statistical significance level was set at 0.05 for all analyses.

## Results

### Population

A total of 142 subjects underwent surgery for AIS during the study period. Eighty subjects had to be excluded for several reasons, as shown in Table 1. Ultimately, 62 AIS patients with full documentation were left for the purpose of this study. On average, the subjects were 15.6 ± 2.5 years of age, 56 (90%) were girls, and most of the curves were classified as type Lenke 1 of these moderate to severe AIS patients (thoracic Cobb angle 37°–110°, lumbar Cobb angle 18°–82°; Table 1).

**Table 1:** Demographics are shown for all included AIS patients and controls. Also, the excluded patients are shown

Demographic parameter		*n* = 62
Age at radiograph (years)	Range	10–23
	Mean ± sd	15.6 ± 2.5
Girls, *n* (%)		56 (90.3%)
Right convexity of main thoracic curve, *n* (%)	Right convex	62 (100%)
Interval CT–radiograph (days)	Range	−7 to 130
	Mean ± sd	2.98 ± 17.2
Interval radiograph–MRI (days)	Range	−46 to 181
	Mean ± sd	81.3 ± 51.4
Interval CT–MRI (days)	Range	−26 to 181
	Mean ± sd	84.2 ± 47.1
Lenke curve type		
I		26
II		12
III		6
IV		4
V		5
VI		9
Exclusion criteria		n
Scan interval >6 months		38
No MRI available		14
No CT scan available		10
Incomplete radiologic work-up		1
Associated congenital or neuromuscular pathologies		12
Left convex main thoracic curve		4
Prior spinal surgery		1

*sd* standard deviation

### Coronal parameters

**Fig. 3:**
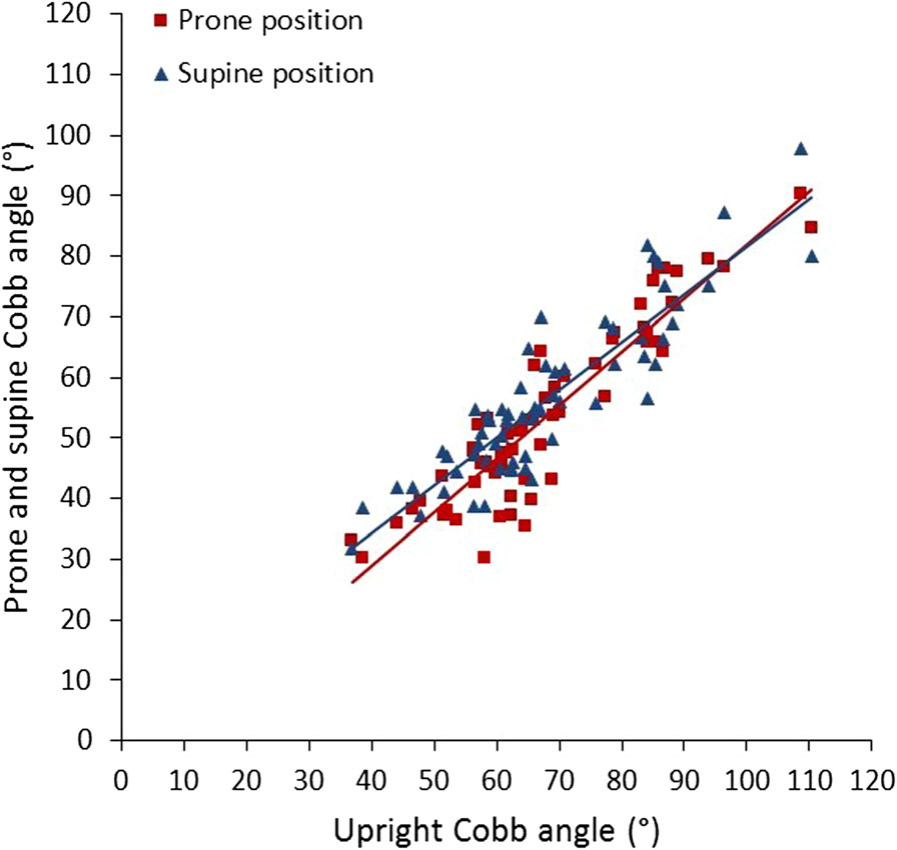
In these scatterplots, the relation between thoracic Cobb angle in the upright, prone (*red trend line*), and supine (*blue trend line*) positions is shown. Although the upright Cobb angle was significantly larger, significant linear correlations were found (ICC 0.967; *P* < 0.001), indicating that with increasing Cobb angle, differences between the body positions increased simultaneously

In the coronal plane, the main thoracic Cobb angle was on average 68° ± 15°, 54° ± 15°, and 57° ± 14° on the upright radiographs, prone CT, and supine MRI, respectively, and differed significantly between all the three positions (*P* < 0.001; Table 2). The average (thoraco)lumbar Cobb angle on the conventional upright radiograph was 44° ± 17° as compared to those on the prone CT (33° ± 15°) and supine MRI (35° ± 16°) (*P* ≤ 0.018, between the three positions). Although the upright angles were larger, the Cobb angles correlated very well between the three positions (ICC: thoracic 0.97 and lumbar 0.96; Table 3; Fig. 3). Significant linear correlations were found, indicating that with increasing Cobb angle, differences between the body positions increased simultaneously. The conversion equations that resulted from the correlation analyses of the different parameters between the upright X-ray, prone CT scan, and supine MRI could be used for conversion purposes (Table 4).

**Table 2:** Differences (mean ± standard deviation) between upright (X), prone (CT), and supine (MRI) positions for Cobb angle, thoracic kyphosis, lumbar lordosis, and apical vertebral rotation in the thoracic as well as lumbar curves. According to the Bland-Altman plot, the *P* value showed if there is agreement by using the *t* test. If this test showed no significant different (*P* > 0.05), a regression analysis was performed to see is if there is agreement, written in brackets

	Upright	Prone	Supine	*P* value		
				X vs. CT	X vs. MRI	CT vs. MRI
Thoracic						
Cobb (°)	68.2 ± 15.4	53.9 ± 14.8	56.7 ± 13.5	<0.001	<0.001	<0.001
Kyphosis (°)	25.8 ± 11.4	22.4 ± 11.6	17.3 ± 9.8	0.004	<0.001	<0.001
Vertebral rotation (°)	21.6 ± 11.7	19.9 ± 8.9	16.3 ± 10.8	0.161 (0.007)	0.001	0.002
Lumbar						
Cobb (°)	44.3 ± 16.8	33.1 ± 15.0	35.2 ± 15.9	<0.001	<0.001	0.018
Lordosis (°)	48.8 ± 12.0	45.4 ± 10.8	43.7 ± 12.4	0.006	<0.001	0.341 (0.620)^a^
Vertebral rotation (°)	10.7 ± 12.8	7.5 ± 11.4	6.2 ± 13.7	0.428 (<0.001)	0.663 (0.129)^a^	0.679 (0.006)

^a^Agreement according to the Bland-Altman plot

**Table 3:** Two-way mixed intraclass correlation coefficient (ICC) and 95% confidence interval (CI) between upright, prone, and supine positions

	ICC (95% CI)	*P* value
Thoracic Cobb angle	0.967 (0.950–0.979)	<0.001
Lumbar Cobb angle	0.964 (0.945–0.977)	<0.001
Thoracic kyphosis	0.873 (0.806–0.919)	<0.001
Lumbar lordosis	0.854 (0.777–0.907)	<0.001
Thoracic apical rotation	0.815 (0.718–0.882)	<0.001
Lumbar apical rotation	0.900 (0.848–0.937)	<0.001

**Table 4:** For translational purposes, the conversion equations that resulted from the linear correlation analyses of the different parameters between the upright X-ray, prone CT scan, and supine MRI are provided for the thoracic (Th) and lumbar (L) Cobb angles

		Cobb angle		
		Upright X-ray	Prone CT scan	Supine MRI
Cobb angle	Upright X-ray	–	Th: CT (°) = −6.2 + 0.88 * X-ray (°) L: CT (°) = −2.7 + 0.81 * X-ray (°)	Th: MRI (°) = 2.9 + 0.79 * X-ray (°) L: MRI (°) = −2.1 + 0.85 * X-ray (°)
	Prone CT	Th: X-ray (°) = 16.6 + 0.96 * CT (°) L: X-ray (°) = 11.1 + 1.00 * CT (°)	–	Th: MRI (°) = 11.0 + 0.85 * CT (°) L: MRI (°) = 4.9 + 0.92 * CT (°)
	Supine MRI	Th: X-ray (°) = 10.8 + 1.01 * MRI (°) L: X-ray (°) = 9.5 + 0.98 * MRI (°)	Th: CT (°) = −2.8 + 1.00 * MRI (°) L: CT (°) = 2.6 + 0.86 * MRI (°)	–

### Axial rotation

Parallel to the coronal Cobb angles, in both the thoracic curve and the (thoraco)lumbar curve, the mean apical vertebral rotation was larger in the upright position (Table 2). Significant correlations, however, were observed between the apical rotation as measured using the Perdriolle method on upright radiographs and the rotation on the prone CT and supine MRI (ICC: thoracic 0.82 and lumbar 0.90; Tables 3 and 4).

### Sagittal parameters

Also in the sagittal plane, the TK in the upright position (26° ± 11°) was significantly larger as compared to that in the prone (22° ± 12°) and supine (17° ± 10°; *P* ≤ 0.004) positions. The upright LL (49° ± 12°) was significantly higher as compared to the prone LL (45° ± 11°) and supine LL (44° ± 12°; *P ≤* 0.006). According to the Bland-Altman method, there was agreement between the LL in the supine and prone positions. The TK and the LL correlated well between all the positions (ICC 0.87 and 0.85; Tables 3 and 4).

### Reliability

The ICCs for intra- and interobserver reliabilities of the Cobb angles, TK, LL, and vertebral rotation on the three modalities were all excellent (>0.93 and >0.74, respectively; Table 5).

**Table 5:** Intra- and interobserver reliability analysis and 95% confidence interval

	X-ray		CT scan		MRI scan	
	Intra	Inter	Intra	Inter	Intra	Inter
Thoracic Cobb	0.993 (0.971–0.998)	0.972 (0.888–0.993)	0.997 (0.988–0.999)	0.995 (0.980–0.999)	0.995 (0.982–0.999)	0.974 (0.896–0.994)
Lumbar Cobb	0.999 (0.996–1.00)	0.995 (0.980–0.999)	0.999 (0.996–1.00)	0.995 (0.981–0.999)	0.997 (0.990–0.999)	0.986 (0.945–0.997)
Thoracic kyphosis	0.989 (0.954–0.997)	0.922 (0.610–0.984)	0.931 (0.722–0.983)	0.864 (0.454–0.966)	0.992 (0.967–0.998)	0.940 (0.759–0.985)
Lumbar lordosis	0.986 (0.944–0.997)	0.989 (0.956–0.997)	0.995 (0.980–0.999)	0.973 (0.890–0.993)	0.995 (0.981–0.999)	0.971 (0.884–0.993)
Thoracic rotation	0.979 (0.915–0.995)	0.977 (0.906–0.994)	^a^	^a^	0.939 (0.756–0.985)	0.744 (0.409–0.964)
Lumbar rotation	0.975 (0.899–0.994)	0.996 (0.985–0.999)	^a^	^a^	0.906 (0.620–0.977)	0.885 (0.539–0.972)

^a^Intra- and interobserver reliability for the rotation on 3-D scans; this method was tested previously (ICC 0.92 and 0.89) [9]

## Discussion

X-rays for scoliosis are, by convention, obtained in an upright position, allowing gravity to have its influence on the morphology of the spine. The drawbacks of this X-ray imaging in analyzing the deformity as well as planning treatment are becoming increasingly clear: the deformity has a complex 3-D nature that is hardly appreciated on plain films, and radiation exposure, even with modern day equipment, is becoming a serious concern. Although the use of ultrasound for diagnosis and follow-up of spinal deformities has been explored and seems promising, this technique gives little detail of the anatomy and needs further evaluation [17–19]. Additional imaging studies are frequently obtained in scoliosis; CT scanning is still considered the gold standard for providing accurate and detailed information on bony anatomy (for instance, in cases where congenital malformations are suspected) and can give accurate 3-D reconstructions of complex deformities [10]. However, CT carries even more radiation exposure and is performed non-weight bearing [10]. MRI is safe, provides accurate information on the spinal cord and other soft tissues, but is also (usually) performed in a non-weight-bearing manner, and is known to show less detail of bony structures. Therefore, it is important to define where these techniques overlap, in order to reduce costs and radiation exposure. Previous studies have already described the differences in morphology of the spine in AIS between different imaging methods and between different body positions [20–26]. This study is, however, to the best of our knowledge, the first to look into the relationship between the three different positions in all three planes of the body to visualize the scoliotic spine.

In this study, we observed that there is underestimation of the deformation of the spine in the supine and prone positions as compared to that in the upright position, which is overall more pronounced in the thoracic curves as compared to the (thoraco)lumbar curves. The lying positions underestimated the thoracic and (thoraco)lumbar Cobb angles for 12°–14° and 9°–11°, respectively; the TK and LL for 3°–9° and 3°–5°, respectively; and the thoracic and lumbar apical vertebral rotations for 2°–5° and 3°–5°, respectively. Therefore, the parameters on supine and prone scans could not directly be compared to the upright radiographs. However, good and excellent linear correlations were observed for the morphological parameters in the coronal (ICC ≥0.964), sagittal (ICC ≥0.854), and axial (ICC ≥0.815) planes between X-ray, CT, and MRI. This implies that reliable conversion of the parameters between the different positions is possible. A limitation of this study is the population that only includes relatively severe curves. From our results, the reliability of conversion of parameters between different positions for patients with mild AIS curves cannot be derived. Shi et al. described the correlation of the coronal Cobb angle between upright and supine positions in mild, moderate, and severe AIS patients and concluded that the correlation coefficients were more reliable in the severe group, probably due to the reduced curve flexibility in the severe group [26, 27]. As we demonstrated before, evaluation of the true sagittal plane in scoliosis on plain X-rays is notoriously unreliable and differs greatly from the true sagittal plane as may be analyzed more accurately on both CT and MRI [28].

## Conclusions

There is a good to excellent correlation of the morphology of the scoliotic spine in all three planes between standard upright X-ray, MRI, and CT scan in these moderate to severe AIS patients. Apparently, at least part of the information obtained by these different modalities overlaps. Findings of this study suggest that severity of scoliotic deformity in AIS patients can be largely represented by different imaging modalities despite the differences in body position. Future longitudinal studies to demonstrate the practical implications of these findings are planned.

## Abbreviations

3-D: Three-dimensional
AIS: Adolescent idiopathic scoliosis
CI: Confidence interval
CT: Computed tomography
DRR: Digital reconstructed radiograph
ICC: Intraclass correlation coefficient
LL: Lumbar lordosis
MRI: Magnetic resonance imaging
PACS: Picture archiving and communications system
sd: Standard deviation
TK: Thoracic kyphosis
